# Veterans Helping Veterans: Reflections on 10 Years of Community Engagement

**DOI:** 10.1007/s11606-021-06986-0

**Published:** 2022-03-29

**Authors:** Ray Facundo

**Affiliations:** Southeast Louisiana Veterans Health Care System, US Department of Veteran Affairs, New Orleans, LA USA

I have always felt that connecting with others in my community would give me the support structure needed to succeed—both personally and professionally. Today, I am a research project manager in Veterans Affairs (VA), a social worker, and an Army veteran—three pieces of my identity that help me quickly connect to my community and the veterans within it. Building relationships with these folks gives me strength, and I hope to always do the same for them.

In 2003, I enlisted and served in the Field Artillery for three years on active duty and deployed for 15 months as a truck driver. Shortly after returning home, I reenlisted with the New Jersey National Guard and deployed again for another year as an aide to the commanding general of detainee operations in Baghdad, Iraq. I’ve spent every year, post-service, working to help my fellow veterans make sense of the VA, a system that was built exclusively to care for them for the remainder of their lives. As a social worker, I see how the barriers within their healthcare system can help them or harm them. As a vet, I draw on personal experience to empathize with them. As a researcher, I dive deeper into those barriers and try to help break them down. Like many veterans before and after me, my desire to help other vets have a better experience drives my efforts to ensure we do our best for them.

**Fig. 1:**
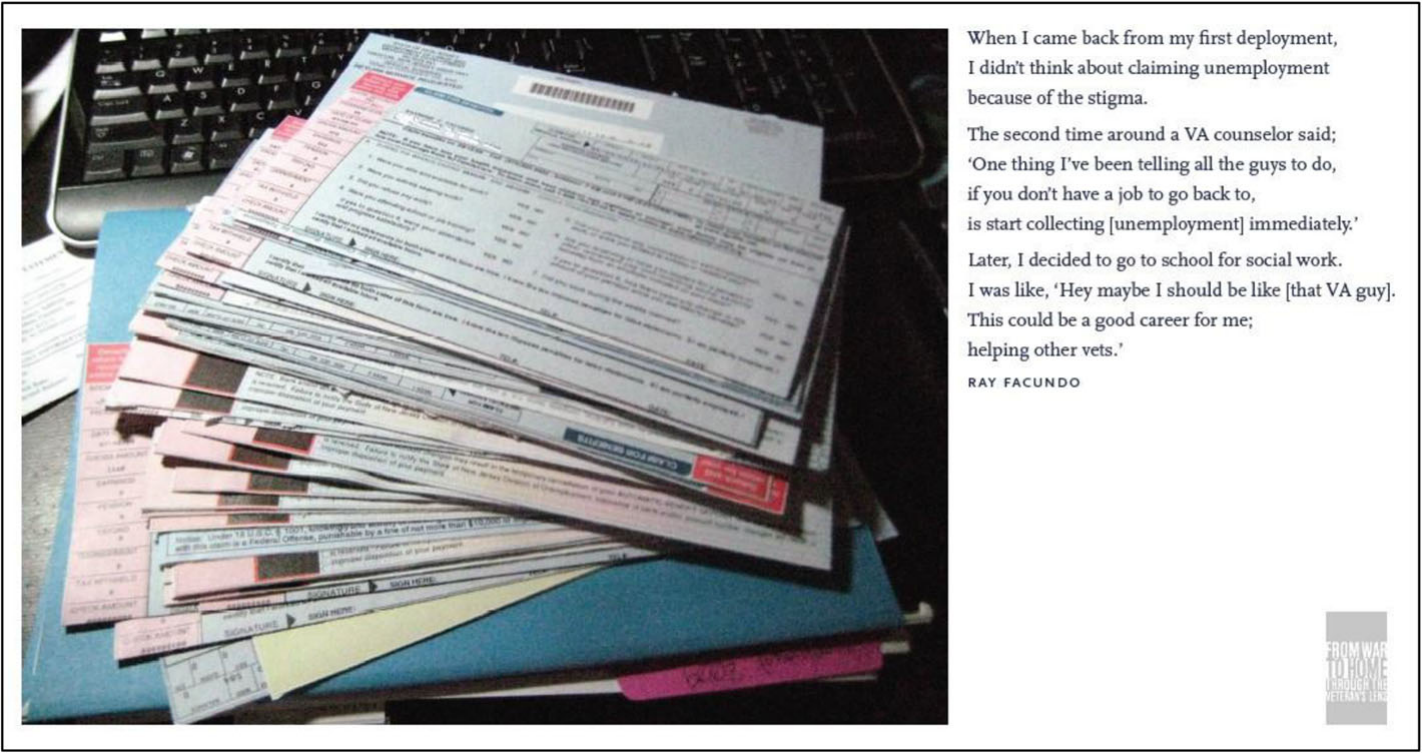
Photo-narrative from *From War to Home: Through the Veterans Lens*, describing one of the challenges many veterans face during reintegration.

Figure 1 is a photo of an exhibition piece from *From War to Home: Through the Veterans Lens*—a photovoice project that described the challenges of transition from military to civilian life. I took a picture of the two years of weekly unemployment I collected when I left the military in 2009, just one year after the recession. I wanted other Veterans to take advantage of this benefit instead of struggling to pay bills, like I did after completing my first tour of duty in 2006. Participating in *From War to Home* was my first experience with community-engaged research, and it gave me a platform to advocate for others like me. I first saw the flyer on the director’s desk at the West Chester University Veterans Center in 2011. He usually tossed flyers from the VA into the trash, but this time, he vouched for the researcher, saying he’d “grilled her for hours and she was legit.” If the researcher had not taken the time to explain the study to him, and he couldn’t see the impact, he would have never displayed a flyer for her. He built the center to support veterans and provide a safe space for them. The flyer mentioned compensation and a free camera, which appealed to me as a broke college student, but without hearing his perspective, I probably would have dismissed it.

I expressed my interest over a short phone call and Dr. Gala True drove from Philadelphia to my home in Delaware, where we spent hours going over consent forms and talking about the project. *From War to Home* was a public-facing advocacy project. The data collected comprised a traveling exhibition of photos with corresponding quotes from photo-elicitation interviews. Gala made it clear that my words and photos would be displayed at many public-facing venues across the United States, including many VA Medical Centers. I had participated in other studies where I had never seen the results, or if what I contributed had made any kind of impact. The photovoice project felt very different.

For many years, Gala kept in touch and asked me to speak on webinars and travel for conferences to help present *From War to Home*. At first, I talked about my experiences in the project, how it was similar to art or narrative therapy, and how it helped me talk about things that I previously had trouble expressing. As my career and my experience as a veteran evolved, the conversations began to shift into why collaborating with VA researchers was important. Veterans needed a stronger voice in the research that affected their care. Researchers were having trouble making connections to veterans outside the VA. I found myself speaking more about accessing the veteran community outside of VHA rosters and how best to approach them.

The work I did with *From War to Home* helped me think about how I could make a broader impact through community engagement and research. Shortly after the photovoice project was first exhibited, I accepted a job at Portland State University to build and direct their new Veterans Resource Center. During my years there, we enrolled up to 1100 unique veterans annually, most of whom had just left the military and were using their education benefits. Aside from my advising duties, my students and I created a needs assessment for our community and collaborated with other student centers to increase diversity and inclusion in our space. I made it my mission to educate the public about the war and the value of the people who experienced it—in the classroom, on the stage, on the radio, or through community service. I hosted storytelling and art-making workshops, developed a peer-mentor program, and networked with every service aimed at veterans in the Portland metro area. Eventually, we created a space for VA to come to us—a closet in our student lounge that we turned into an office for our visiting VA Vocational Rehabilitation counselors. On the days they weren’t on campus, we opened the office to other veteran service officers and VA researchers looking to connect to our students. When researchers approached me, I asked them to first build trust with us. Instead of hanging flyers, we invited researchers to present information about their studies and gave them time to answer questions from veterans.

In 2014, Dr. Sarah Ono, a research investigator at VA, asked me to be a part of her seed committee for a new veteran engagement group, and I helped her identify some members who I believe are still advising research today. From working with Gala and Sarah, I saw a lot of the research being conducted as beneficial to the veteran community, so I took on the work of connecting them.

In 2015, I was co-presenting with Gala and Sarah at the VA Health Sciences Research and Development Quality Enhancement Research Initiative National Meeting, and Gala told me she was relocating to New Orleans and starting another 3-year photovoice project. I asked if she needed a project manager, and soon enough, we began the year -long process of hiring and onboarding. Upon arriving in New Orleans, I began identifying veteran organizations and started meeting with whoever would give me the time. I volunteered. I helped create and organize events for veterans, their friends and families. I showed up for meetings. We get to meet a lot of people through community-engaged research, so we create opportunities to connect. In my experience, most communities of veterans grew organically, and friendships were formed from common experience and storytelling.

A lot of the folks who collaborated on the projects I worked on became friends. We don’t just eat together—we have co-authored a paper with Veterans and caregivers that appeared in a special issue of the Journal of Humanistic Psychology.^1^ We have another paper, co-authored with veterans with a traumatic brain injury, that is forthcoming in a special issue of the Journal of Community Engagement and Scholarship.^2^ They serve on our advisory boards and continue to connect us to other vets in their communities. We continue to call on them when we need their voices, and they always answer.

“Veterans helping veterans” has been the mantra of almost every veteran-focused organization I’ve worked with over the past decade, and I have heard many folks say that we are the only ones who can truly understand other veterans. The American Legion, Veterans of Foreign Wars, and similar groups seem like the most common places that researchers look for veterans—but there don’t seem to be many researchers in the ranks of their membership. If the VA could hire more veterans into research roles, and prioritize engagement with these groups, we may be able to make a bigger impact. They could easily protect time for veterans working in research to be liaisons and build bridges to groups like The Mission Continues, Team Rubicon, Veterans For Peace, About Face, and other veterans groups who could broaden the spectrum of diversity and inclusion. The future of VA research depends on VA’s commitment to support and push collaborative health services research forward. Together, veterans, and veteran or civilian researchers can truly break new scientific ground, not only for the betterment of VA health care, but potentially for health care systems in general.
